# Supplementary material for: Modeling glioblastoma heterogeneity as a dynamic network of cell states
Source: Mol Syst Biol. 2021 Sep 16;17(9):e10105. doi: 10.15252/msb.202010105 (PMC8444284; doi:10.15252/msb.202010105)
Supplement: Supplementary file 6 — Source Data for Figure 5 [file MSB-17-e10105-s004.zip › Figure5A_sourcedata/GSEA_3071/nosigdb_stateIV.GseaPreranked.1622795164626/RICHARDS_NOWAKOWSKI_SCIENCE_2017_OPC_UPREG.html]

Details for gene set RICHARDS\_NOWAKOWSKI\_SCIENCE\_2017\_OPC\_UPREG[GSEA]

|  || Dataset | state53071 |
| Phenotype | NoPhenotypeAvailable |
| Upregulated in class | na\_pos |
| GeneSet | RICHARDS\_NOWAKOWSKI\_SCIENCE\_2017\_OPC\_UPREG |
| Enrichment Score (ES) | 0.51428914 |
| Normalized Enrichment Score (NES) | 3.2597592 |
| Nominal p-value | 0.0 |
| FDR q-value | 0.0 |
| FWER p-Value | 0.0 |
Table: GSEA Results Summary

  

Fig 1: Enrichment plot: RICHARDS\_NOWAKOWSKI\_SCIENCE\_2017\_OPC\_UPREG      
 Profile of the Running ES Score & Positions of GeneSet Members on the Rank Ordered List

  

| PROBE | GENE SYMBOL | GENE\_TITLE | RANK IN GENE LIST | RANK METRIC SCORE | RUNNING ES | CORE ENRICHMENT || 1 | PDGFRA |  |  | 1 | 2.924 | 0.0150 | Yes |
| 2 | C1orf61 |  |  | 3 | 2.760 | 0.0291 | Yes |
| 3 | PTPRZ1 |  |  | 10 | 2.539 | 0.0411 | Yes |
| 4 | PMP2 |  |  | 11 | 2.494 | 0.0541 | Yes |
| 5 | S100B |  |  | 12 | 2.461 | 0.0669 | Yes |
| 6 | GPM6B |  |  | 21 | 2.210 | 0.0768 | Yes |
| 7 | PTN |  |  | 25 | 2.140 | 0.0873 | Yes |
| 8 | SCRG1 |  |  | 28 | 2.056 | 0.0976 | Yes |
| 9 | LRRN3 |  |  | 29 | 2.015 | 0.1081 | Yes |
| 10 | MAP3K1 |  |  | 42 | 1.903 | 0.1156 | Yes |
| 11 | CADM4 |  |  | 47 | 1.860 | 0.1245 | Yes |
| 12 | MAP2 |  |  | 55 | 1.811 | 0.1325 | Yes |
| 13 | LIMA1 |  |  | 58 | 1.805 | 0.1415 | Yes |
| 14 | SLC35F1 |  |  | 78 | 1.647 | 0.1463 | Yes |
| 15 | ITPR2 |  |  | 88 | 1.610 | 0.1529 | Yes |
| 16 | MMP16 |  |  | 90 | 1.578 | 0.1609 | Yes |
| 17 | GPM6A |  |  | 98 | 1.538 | 0.1675 | Yes |
| 18 | SPRY1 |  |  | 103 | 1.523 | 0.1746 | Yes |
| 19 | MYO10 |  |  | 121 | 1.484 | 0.1789 | Yes |
| 20 | LRRN1 |  |  | 122 | 1.482 | 0.1867 | Yes |
| 21 | FGFBP3 |  |  | 127 | 1.465 | 0.1935 | Yes |
| 22 | PROM1 |  |  | 128 | 1.462 | 0.2011 | Yes |
| 23 | LSAMP |  |  | 130 | 1.454 | 0.2084 | Yes |
| 24 | CHD7 |  |  | 144 | 1.386 | 0.2131 | Yes |
| 25 | SULF2 |  |  | 146 | 1.384 | 0.2201 | Yes |
| 26 | TMEFF2 |  |  | 152 | 1.370 | 0.2262 | Yes |
| 27 | BCHE |  |  | 155 | 1.362 | 0.2329 | Yes |
| 28 | DUSP6 |  |  | 156 | 1.359 | 0.2399 | Yes |
| 29 | CDH13 |  |  | 158 | 1.349 | 0.2467 | Yes |
| 30 | GRIA2 |  |  | 160 | 1.347 | 0.2535 | Yes |
| 31 | IL1RAP |  |  | 171 | 1.317 | 0.2584 | Yes |
| 32 | ETV5 |  |  | 174 | 1.305 | 0.2648 | Yes |
| 33 | ZNF385D |  |  | 177 | 1.303 | 0.2712 | Yes |
| 34 | CDCA7L |  |  | 185 | 1.292 | 0.2765 | Yes |
| 35 | LCORL |  |  | 186 | 1.289 | 0.2832 | Yes |
| 36 | SOX6 |  |  | 207 | 1.244 | 0.2857 | Yes |
| 37 | SOX2 |  |  | 212 | 1.231 | 0.2913 | Yes |
| 38 | SHC3 |  |  | 215 | 1.218 | 0.2972 | Yes |
| 39 | SATB1 |  |  | 222 | 1.203 | 0.3023 | Yes |
| 40 | GPR155 |  |  | 227 | 1.191 | 0.3077 | Yes |
| 41 | FABP5 |  |  | 230 | 1.187 | 0.3135 | Yes |
| 42 | TNFRSF21 |  |  | 232 | 1.184 | 0.3194 | Yes |
| 43 | ITM2B |  |  | 233 | 1.181 | 0.3256 | Yes |
| 44 | NOVA1 |  |  | 234 | 1.181 | 0.3317 | Yes |
| 45 | ERBB3 |  |  | 243 | 1.164 | 0.3362 | Yes |
| 46 | KCNQ1OT1 |  |  | 246 | 1.159 | 0.3418 | Yes |
| 47 | SEMA5A |  |  | 247 | 1.153 | 0.3478 | Yes |
| 48 | LITAF |  |  | 252 | 1.144 | 0.3530 | Yes |
| 49 | WSCD1 |  |  | 253 | 1.141 | 0.3589 | Yes |
| 50 | PCDHGC3 |  |  | 255 | 1.133 | 0.3646 | Yes |
| 51 | GNB4 |  |  | 256 | 1.133 | 0.3705 | Yes |
| 52 | PCMTD2 |  |  | 278 | 1.078 | 0.3719 | Yes |
| 53 | PLAT |  |  | 281 | 1.070 | 0.3771 | Yes |
| 54 | NLGN1 |  |  | 282 | 1.069 | 0.3826 | Yes |
| 55 | NLGN4X |  |  | 284 | 1.068 | 0.3880 | Yes |
| 56 | CD9 |  |  | 288 | 1.062 | 0.3929 | Yes |
| 57 | PCDH9 |  |  | 301 | 1.034 | 0.3959 | Yes |
| 58 | SPRY4 |  |  | 304 | 1.030 | 0.4009 | Yes |
| 59 | SALL3 |  |  | 306 | 1.029 | 0.4060 | Yes |
| 60 | ITGB8 |  |  | 307 | 1.027 | 0.4113 | Yes |
| 61 | CACNB2 |  |  | 327 | 0.992 | 0.4127 | Yes |
| 62 | ST3GAL5 |  |  | 336 | 0.985 | 0.4163 | Yes |
| 63 | LYPD1 |  |  | 338 | 0.980 | 0.4212 | Yes |
| 64 | DBI |  |  | 339 | 0.980 | 0.4263 | Yes |
| 65 | MAGI1 |  |  | 355 | 0.969 | 0.4283 | Yes |
| 66 | CALCRL |  |  | 362 | 0.953 | 0.4321 | Yes |
| 67 | BAALC |  |  | 367 | 0.944 | 0.4362 | Yes |
| 68 | EMC10 |  |  | 368 | 0.943 | 0.4411 | Yes |
| 69 | CTTNBP2 |  |  | 369 | 0.942 | 0.4460 | Yes |
| 70 | ZEB1 |  |  | 385 | 0.920 | 0.4478 | Yes |
| 71 | OLIG1 |  |  | 391 | 0.914 | 0.4516 | Yes |
| 72 | TRIB2 |  |  | 396 | 0.911 | 0.4555 | Yes |
| 73 | ATCAY |  |  | 397 | 0.910 | 0.4602 | Yes |
| 74 | SCARB2 |  |  | 426 | 0.876 | 0.4593 | Yes |
| 75 | RFTN2 |  |  | 432 | 0.868 | 0.4628 | Yes |
| 76 | PLEKHH2 |  |  | 443 | 0.860 | 0.4653 | Yes |
| 77 | LAPTM4B |  |  | 468 | 0.843 | 0.4649 | Yes |
| 78 | IDH1 |  |  | 469 | 0.842 | 0.4693 | Yes |
| 79 | MAML2 |  |  | 470 | 0.841 | 0.4736 | Yes |
| 80 | ETV1 |  |  | 488 | 0.824 | 0.4746 | Yes |
| 81 | DSEL |  |  | 506 | 0.812 | 0.4754 | Yes |
| 82 | ABAT |  |  | 515 | 0.805 | 0.4780 | Yes |
| 83 | PDE4B |  |  | 519 | 0.798 | 0.4816 | Yes |
| 84 | TNK2 |  |  | 522 | 0.795 | 0.4853 | Yes |
| 85 | DAG1 |  |  | 525 | 0.794 | 0.4890 | Yes |
| 86 | AASS |  |  | 546 | 0.770 | 0.4891 | Yes |
| 87 | SCD5 |  |  | 555 | 0.759 | 0.4914 | Yes |
| 88 | SPATA6 |  |  | 559 | 0.756 | 0.4948 | Yes |
| 89 | TENM1 |  |  | 560 | 0.756 | 0.4987 | Yes |
| 90 | HIP1 |  |  | 563 | 0.753 | 0.5022 | Yes |
| 91 | FAM84A |  |  | 569 | 0.750 | 0.5051 | Yes |
| 92 | GLCCI1 |  |  | 611 | 0.719 | 0.5008 | Yes |
| 93 | SCG3 |  |  | 632 | 0.707 | 0.5005 | Yes |
| 94 | ILDR2 |  |  | 637 | 0.705 | 0.5033 | Yes |
| 95 | CSGALNACT1 |  |  | 660 | 0.681 | 0.5025 | Yes |
| 96 | STK32A |  |  | 671 | 0.676 | 0.5041 | Yes |
| 97 | SETD5 |  |  | 681 | 0.672 | 0.5058 | Yes |
| 98 | SLC2A13 |  |  | 693 | 0.663 | 0.5070 | Yes |
| 99 | PCBP4 |  |  | 719 | 0.649 | 0.5055 | Yes |
| 100 | ITM2C |  |  | 724 | 0.645 | 0.5080 | Yes |
| 101 | TMX3 |  |  | 751 | 0.633 | 0.5062 | Yes |
| 102 | TMEM132B |  |  | 757 | 0.625 | 0.5084 | Yes |
| 103 | SLC22A17 |  |  | 762 | 0.623 | 0.5109 | Yes |
| 104 | GOLIM4 |  |  | 771 | 0.619 | 0.5125 | Yes |
| 105 | LRRTM3 |  |  | 782 | 0.613 | 0.5137 | Yes |
| 106 | GLTP |  |  | 796 | 0.604 | 0.5143 | Yes |
| 107 | SEMA6D |  |  | 830 | 0.589 | 0.5108 | No |
| 108 | SAMD8 |  |  | 841 | 0.584 | 0.5119 | No |
| 109 | RAB31 |  |  | 864 | 0.574 | 0.5105 | No |
| 110 | SPRY2 |  |  | 871 | 0.573 | 0.5123 | No |
| 111 | SLITRK2 |  |  | 889 | 0.565 | 0.5119 | No |
| 112 | HIPK2 |  |  | 896 | 0.563 | 0.5136 | No |
| 113 | MT3 |  |  | 913 | 0.555 | 0.5133 | No |
| 114 | PCDH17 |  |  | 956 | 0.541 | 0.5078 | No |
| 115 | ZMAT3 |  |  | 966 | 0.536 | 0.5088 | No |
| 116 | PHLPP1 |  |  | 977 | 0.533 | 0.5096 | No |
| 117 | PGRMC1 |  |  | 1004 | 0.521 | 0.5072 | No |
| 118 | HAS2 |  |  | 1066 | 0.502 | 0.4977 | No |
| 119 | QKI |  |  | 1078 | 0.500 | 0.4982 | No |
| 120 | SKIL |  |  | 1091 | 0.495 | 0.4983 | No |
| 121 | ZNF462 |  |  | 1111 | 0.488 | 0.4971 | No |
| 122 | OLIG2 |  |  | 1129 | 0.486 | 0.4963 | No |
| 123 | GATM |  |  | 1148 | 0.481 | 0.4952 | No |
| 124 | ARNT2 |  |  | 1149 | 0.480 | 0.4977 | No |
| 125 | PHLDB1 |  |  | 1168 | 0.475 | 0.4966 | No |
| 126 | TP53INP1 |  |  | 1174 | 0.473 | 0.4981 | No |
| 127 | TSPAN7 |  |  | 1181 | 0.471 | 0.4993 | No |
| 128 | CHST3 |  |  | 1185 | 0.470 | 0.5012 | No |
| 129 | FGF14 |  |  | 1186 | 0.470 | 0.5036 | No |
| 130 | PRKCA |  |  | 1214 | 0.461 | 0.5007 | No |
| 131 | CLDND1 |  |  | 1224 | 0.458 | 0.5013 | No |
| 132 | CPEB2 |  |  | 1256 | 0.451 | 0.4975 | No |
| 133 | PHYHIPL |  |  | 1270 | 0.447 | 0.4973 | No |
| 134 | CFLAR |  |  | 1341 | 0.430 | 0.4856 | No |
| 135 | BMP7 |  |  | 1368 | 0.423 | 0.4827 | No |
| 136 | FOXN2 |  |  | 1381 | 0.421 | 0.4825 | No |
| 137 | SERINC5 |  |  | 1383 | 0.421 | 0.4845 | No |
| 138 | TMEM206 |  |  | 1393 | 0.418 | 0.4849 | No |
| 139 | LRP1B |  |  | 1395 | 0.417 | 0.4869 | No |
| 140 | KIZ |  |  | 1402 | 0.416 | 0.4878 | No |
| 141 | ANGPTL2 |  |  | 1420 | 0.412 | 0.4866 | No |
| 142 | C2orf80 |  |  | 1447 | 0.403 | 0.4836 | No |
| 143 | FYN |  |  | 1483 | 0.394 | 0.4787 | No |
| 144 | KCND3 |  |  | 1539 | 0.381 | 0.4698 | No |
| 145 | GRIA4 |  |  | 1545 | 0.379 | 0.4708 | No |
| 146 | EDNRB |  |  | 1549 | 0.378 | 0.4721 | No |
| 147 | CNP |  |  | 1575 | 0.371 | 0.4691 | No |
| 148 | CHRM3 |  |  | 1662 | 0.357 | 0.4539 | No |
| 149 | DENND5A |  |  | 1673 | 0.355 | 0.4538 | No |
| 150 | MARCKS |  |  | 1713 | 0.348 | 0.4479 | No |
| 151 | LRRC4C |  |  | 1737 | 0.342 | 0.4451 | No |
| 152 | WDR83OS |  |  | 1761 | 0.337 | 0.4423 | No |
| 153 | NLGN3 |  |  | 1785 | 0.333 | 0.4395 | No |
| 154 | CHIC2 |  |  | 1819 | 0.328 | 0.4347 | No |
| 155 | ASIC1 |  |  | 1838 | 0.326 | 0.4328 | No |
| 156 | YIPF6 |  |  | 1896 | 0.315 | 0.4232 | No |
| 157 | RAP2A |  |  | 1903 | 0.314 | 0.4236 | No |
| 158 | OCIAD2 |  |  | 1906 | 0.313 | 0.4248 | No |
| 159 | PELI1 |  |  | 1921 | 0.310 | 0.4237 | No |
| 160 | NXPH1 |  |  | 1950 | 0.306 | 0.4197 | No |
| 161 | CASK |  |  | 2014 | 0.294 | 0.4088 | No |
| 162 | SEPT2 |  |  | 2017 | 0.294 | 0.4099 | No |
| 163 | SHISA9 |  |  | 2019 | 0.293 | 0.4113 | No |
| 164 | SEZ6L |  |  | 2065 | 0.287 | 0.4038 | No |
| 165 | GLDC |  |  | 2077 | 0.286 | 0.4032 | No |
| 166 | SEPT8 |  |  | 2115 | 0.282 | 0.3973 | No |
| 167 | RAB9A |  |  | 2129 | 0.280 | 0.3962 | No |
| 168 | PON2 |  |  | 2177 | 0.271 | 0.3883 | No |
| 169 | PTPRE |  |  | 2187 | 0.269 | 0.3879 | No |
| 170 | XYLT1 |  |  | 2244 | 0.263 | 0.3782 | No |
| 171 | MYT1 |  |  | 2292 | 0.257 | 0.3702 | No |
| 172 | ZNF37A |  |  | 2301 | 0.255 | 0.3700 | No |
| 173 | CUTC |  |  | 2340 | -0.251 | 0.3638 | No |
| 174 | B2M |  |  | 2349 | -0.252 | 0.3635 | No |
| 175 | PPIB |  |  | 2412 | -0.259 | 0.3526 | No |
| 176 | MRPL32 |  |  | 2461 | -0.264 | 0.3444 | No |
| 177 | AFAP1L2 |  |  | 2488 | -0.268 | 0.3407 | No |
| 178 | FNTA |  |  | 2495 | -0.269 | 0.3409 | No |
| 179 | KIAA0232 |  |  | 2529 | -0.272 | 0.3358 | No |
| 180 | UFL1 |  |  | 2573 | -0.279 | 0.3287 | No |
| 181 | MTPAP |  |  | 2669 | -0.291 | 0.3114 | No |
| 182 | MPZL1 |  |  | 2678 | -0.292 | 0.3114 | No |
| 183 | SEPT7 |  |  | 2703 | -0.295 | 0.3082 | No |
| 184 | GCC2 |  |  | 2818 | -0.308 | 0.2872 | No |
| 185 | SNX7 |  |  | 2827 | -0.309 | 0.2872 | No |
| 186 | KLHL42 |  |  | 2854 | -0.313 | 0.2837 | No |
| 187 | GRIK2 |  |  | 2858 | -0.314 | 0.2847 | No |
| 188 | NUP50 |  |  | 2954 | -0.326 | 0.2676 | No |
| 189 | RIN2 |  |  | 3002 | -0.332 | 0.2601 | No |
| 190 | TJP1 |  |  | 3113 | -0.348 | 0.2401 | No |
| 191 | B9D1 |  |  | 3121 | -0.349 | 0.2405 | No |
| 192 | DENR |  |  | 3129 | -0.351 | 0.2410 | No |
| 193 | MBP |  |  | 3231 | -0.365 | 0.2229 | No |
| 194 | RNF144A |  |  | 3276 | -0.371 | 0.2161 | No |
| 195 | PLOD2 |  |  | 3453 | -0.400 | 0.1834 | No |
| 196 | TCF7L2 |  |  | 3497 | -0.409 | 0.1770 | No |
| 197 | SDC3 |  |  | 3547 | -0.419 | 0.1695 | No |
| 198 | NME1 |  |  | 3548 | -0.419 | 0.1716 | No |
| 199 | HSPA5 |  |  | 3555 | -0.419 | 0.1726 | No |
| 200 | PCDH7 |  |  | 3576 | -0.423 | 0.1709 | No |
| 201 | CISD1 |  |  | 3644 | -0.435 | 0.1599 | No |
| 202 | CPT1A |  |  | 3665 | -0.437 | 0.1582 | No |
| 203 | NTNG1 |  |  | 3719 | -0.449 | 0.1500 | No |
| 204 | PGM3 |  |  | 3726 | -0.450 | 0.1512 | No |
| 205 | STARD4 |  |  | 3785 | -0.463 | 0.1421 | No |
| 206 | EVI2A |  |  | 3809 | -0.468 | 0.1400 | No |
| 207 | NOC3L |  |  | 3978 | -0.505 | 0.1094 | No |
| 208 | ACP1 |  |  | 4084 | -0.535 | 0.0914 | No |
| 209 | BLM |  |  | 4100 | -0.541 | 0.0912 | No |
| 210 | RSU1 |  |  | 4127 | -0.546 | 0.0889 | No |
| 211 | DSTN |  |  | 4204 | -0.569 | 0.0768 | No |
| 212 | BCAT1 |  |  | 4272 | -0.590 | 0.0667 | No |
| 213 | SLC25A5 |  |  | 4296 | -0.597 | 0.0652 | No |
| 214 | ITGAV |  |  | 4312 | -0.601 | 0.0654 | No |
| 215 | SH3D19 |  |  | 4326 | -0.605 | 0.0659 | No |
| 216 | VCAN |  |  | 4464 | -0.661 | 0.0423 | No |
| 217 | GALNT13 |  |  | 4466 | -0.661 | 0.0455 | No |
| 218 | SPCS3 |  |  | 4512 | -0.686 | 0.0402 | No |
| 219 | TNS3 |  |  | 4541 | -0.700 | 0.0383 | No |
| 220 | THY1 |  |  | 4587 | -0.729 | 0.0331 | No |
| 221 | NT5E |  |  | 4607 | -0.737 | 0.0332 | No |
| 222 | PDLIM5 |  |  | 4612 | -0.739 | 0.0363 | No |
| 223 | PKP4 |  |  | 4652 | -0.764 | 0.0325 | No |
| 224 | ARL4A |  |  | 4695 | -0.791 | 0.0283 | No |
| 225 | MYBL1 |  |  | 4697 | -0.795 | 0.0322 | No |
| 226 | APCDD1 |  |  | 4762 | -0.837 | 0.0239 | No |
| 227 | DOCK10 |  |  | 4802 | -0.866 | 0.0207 | No |
| 228 | NDUFAF2 |  |  | 4862 | -0.926 | 0.0138 | No |
| 229 | PDGFA |  |  | 4967 | -1.039 | -0.0013 | No |
| 230 | EPB41L2 |  |  | 4974 | -1.051 | 0.0029 | No |
| 231 | UGDH |  |  | 5034 | -1.164 | -0.0027 | No |
| 232 | NTM |  |  | 5052 | -1.188 | 0.0001 | No |
| 233 | ALCAM |  |  | 5118 | -1.327 | -0.0058 | No |
| 234 | LRP1 |  |  | 5152 | -1.437 | -0.0049 | No |
| 235 | COL11A1 |  |  | 5169 | -1.526 | -0.0001 | No |
| 236 | FRMD6 |  |  | 5195 | -1.640 | 0.0034 | No |
| 237 | EDIL3 |  |  | 5282 | -2.918 | 0.0016 | No |
Table: GSEA details [plain text format]

  

Fig 2: RICHARDS\_NOWAKOWSKI\_SCIENCE\_2017\_OPC\_UPREG: Random ES distribution      
 Gene set null distribution of ES for **RICHARDS\_NOWAKOWSKI\_SCIENCE\_2017\_OPC\_UPREG**
